# Assessing soil system changes under climate‐smart agriculture via farmers' observations and conventional soil testing

**DOI:** 10.1002/ldr.4339

**Published:** 2022-05-31

**Authors:** Samuel Eze, Andrew J. Dougill, Steven A. Banwart, Susannah M. Sallu, Rashid N. Mgohele, Catherine J. Senkoro

**Affiliations:** ^1^ School of Earth and Environment, Faculty of Environment University of Leeds Leeds UK; ^2^ Tanzanian Agricultural Research Institute (TARI), Mlingano Centre Tanga Tanzania

**Keywords:** African highlands, climate resilience, local soil knowledge, soil conservation, soil health, Usambara Mountains

## Abstract

Soil degradation remains a challenge in African highlands, where land management lacks a strong context‐specific evidence base. We investigated the impacts of recently implemented soil and water conservation (SWC) practices—farmyard manure addition, incorporation of crop residues in soil and *fanya juu* terracing under an agroforestry system on soil health indicators in the East Usambara Mountains of Tanzania. Farmers' observations of soil changes were combined with conventional soil testing to assess the initial impacts of SWC practices relative to conventional non‐SWC practice. Majority of farmers (66%–83%) reported that combining *fanya juu* terracing with organic amendments led to soil colour change from red to black and an increase in crop yield. Despite the observed darkening of the soil, there was no significant increase in soil organic carbon stock and the contents of N, P, K. There were important changes in soil physical properties, including greater aggregate stability (mean weight diameter of 1.51–1.71 mm) in the SWC plots, a greater volume of transmission pores (>60 μm) and coarse storage pores (10–60 μm) in the surface soil layer (0–15 cm), and greater volume of fine storage pores (0.2–10 μm) and residual pores (0.2 μm) in the sub‐surface layer (15–30 cm) of the SWC plots compared with the conventional plots. These changes indicate that SWC rapidly enhances infiltration and retention of water within the root zone, which are important for increasing crop yields and improving the resilience of the agro‐ecosystem to environmental stress. Combining SWC with effective soil fertility management is needed for sustainable highland agriculture.

## 1 INTRODUCTION

Soil degradation is a major factor limiting agricultural productivity and food security in sub‐Saharan Africa (SSA) with soil erosion, structural deterioration, organic matter loss and fertility decline being widespread threats to soil functions (FAO and ITPS, 2015). Degradation of 65% of the soils on agricultural lands in Africa occurred in the last century (Oldeman, 1991), and the situation has not improved significantly as annual soil loss of up to 18.4 t ha^−1^ is still being recorded (Fenta et al., 2020). Climate change, via increase in the frequency of intensive/erosive rainfall, is one of the leading contributors to soil degradation and the resulting decline in crop yields across SSA (Stuch et al.,  2021). In highland regions of SSA, steep topography and unsustainable land management practices enhance the acceleration of soil degradation, presenting a bleak future for food security (Fenta et al., 2020).

Reversing soil degradation and enhancing soil health requires changes in existing land management practices. The need to curb soil degradation combined with addressing climate change concerns has led to the promotion of climate‐smart agriculture (CSA) globally and particularly in SSA (Ambaw et al., 2020). CSA refers to a suite of agricultural land management strategies with the triple objectives of “…sustainably increasing agricultural productivity and incomes, enhancing resilience and adaptation to climate change, and reducing greenhouse gas emissions” (Faurès et al., 2013). CSA in highland regions of Africa includes soil and water conservation (SWC) practices such as contour barriers e.g. terracing and planting of grass strips across steep slopes, minimum tillage and soil fertility management practices such as addition of organic manure, with the promise of reducing erosion and greenhouse gas emissions (Ambaw et al., 2020), improving water and nutrient retention, increasing crop yield, reducing labour needs, and increasing the net income of smallholder farmers (Adgo & Akalu, 2010). *Fanya juu* terracing is a key element of SWC in the highlands and an erosion‐control measure that involves digging of trenches across slopes with excavated soils thrown uphill to form an embankment (Chen et al., 2017). Soil health benefits of *fanya juu* terracing are believed to be better achieved through integration with other SWC measures such as agroforestry, addition of farmyard manure, mulching and incorporation of crop residues in the soil (Wolka et al., 2018).

Many factors including lack of land tenure security (Bullock et al., 2014) and high cost of organic manure and labour for constructing cross‐slope barriers such as *fanya juu* terracing (Bizoza & De Graaff, 2012) have been identified as impediments to the adoption of SWC practices among smallholder farmers. There is also a lack of a strong context‐specific evidence base on the impacts of specific SWC practices on soil health, which can limit their adoption among farmers. Without context‐specific evidence on land management impacts, CSA recommendations for one region may rely on results of studies in another region that differs in cultural, socio‐economic, and environmental characteristics, thereby leading to variable results. This impacts negatively on the reliability of information provided to farmers e.g. via agricultural extension services and training, which is an important factor in farmers' adoption of multiple SWC practices (Arslan et al., 2017; Belachew et al., 2020).

To build an evidence base on the impacts of CSA on soil health and provide farmers with relevant information on CSA benefits, most researchers depend only on empirical measurements of changes in soil health indicators such as aggregate stability, porosity, infiltration rate, water holding capacity, organic matter/carbon and nutrient levels (Cardoso et al., 2013). Farmers' experiences in terms of their observed impacts of land management practices on soil systems tend to be ignored and remain unknown in many parts of the African highlands. As farmers tend to draw from their lived experiences and observations in making land management decisions (Hermans et al., 2021), combining conventional soil testing with farmers' observations in an integrative approach will help to build a comprehensive evidence base on the impacts of CSA on soil health.

By combining farmers' observations with conventional soil assessments, this study builds an evidence base on the impacts of CSA on soil health in an important African highland region in the East Usambara Mountains, Tanzania. The aim was to assess the impacts of selected SWC practices on soil health indicators. Specific objectives were to:

1. Assess farmers' understanding of the impacts of farmyard manure (*FYM*) addition alone and in combination with incorporation of crop residues (*CR*) in the soil and *fanya juu* terracing (*Ter*) under agroforestry system on soil health indicators used to guide land management decision‐making.
2. Assess the impacts of farmyard manure addition alone and in combination with incorporation of crop residues in the soil and *fanya juu* terracing under agroforestry system on soil carbon storage, aggregate stability, and concentrations of nitrogen, phosphorus and potassium.
3. Compare the results of empirical soil testing with farmers' observed changes in soil health indicators due to SWC practices after 2–18 years of implementation.

The hypotheses guiding the study are that: (1) combining farmyard manure addition with *fanya juu* terracing under agroforestry system will have greater impacts on soil health than farmyard manure alone; (2) results of conventional soil testing will match farmers' observed impacts of SWC on the soil system.

## 2 METHODOLOGY

### 2.1 Study area

The study was conducted in three villages (Kwemsoso, Mgambo and Misalai) between latitudes 5°0′–5°2′S and longitudes 38°36′–38°36′E, within the East Usambara Mountains (EUM) located in Tanga region of northeastern Tanzania. The EUM forms part of the Eastern Arc Mountains, a chain of high biodiversity tropical mountain ecosystems that stretches from Udzungwa Mountains in south‐central Tanzania to Taita Hills in southeastern Kenya. Communities in the EUM are interspersed between tea estates and forest reserves and rely on subsistence agriculture. Food crops include maize, cassava and beans, whereas cash crops are mainly spices such as cardamom (*Elettaria cardamomum* [L.] Maton.), cinnamon (*Cinnamomum verum* J. Presl.), cloves (*Syzygium aromaticum* [L.] Merr.) and black pepper (*Piper nigrum* L.), which are grown in agroforestry systems (Powell et al., 2013).

Our study focused on the EUM highlands that are 900 m or more above sea level. Environmental features of these highlands including soils, vegetation and climate differ from those in lower altitudes and require different land management practices (Hamilton, 1998). The highlands in the EUM are characterized by steep slopes of 15%–50%, with Acrisols or Ferralsols (World Reference Base, 2015) consisting of mainly low‐activity clay minerals such as kaolinites and sesquioxides (Kirsten et al., 2016). Soil erosion by water is a major environmental problem in the EUM and other Tanzanian highlands with an annual soil loss of up to 10.1 t ha^−1^ in croplands, which is much higher than the average soil loss of 6.3 t ha^−1^ yr^−1^ in the East African region (Fenta et al., 2020). To address the soil degradation problems and enhance food security, on‐farm climate‐smart land management practices have been promoted in some villages in the EUM as part of an European Union's Global Climate Change Alliance (GCCA+) integrated adaptation programme (European Union, 2019). On‐farm SWC practices introduced and promoted through farmer‐field schools on fields that were previously under traditional tillage systems include construction of *fanya juu* terraces and use of grass strips to stabilize the soil, agroforestry and addition of organic manure, diversification of crops to include perennial spices and planting of drought tolerant maize varieties. These practices formed the basis of this study.

### 2.2 Research design

Farmer's reported soil changes were combined with conventional soil testing in a hybrid approach (Hermans et al., 2021). This approach combines the advantages of both qualitative and quantitative soil assessment techniques. Farmer's visual techniques helped in understanding land management impacts on soils from the land managers' perspective rather than relying on only laboratory techniques which provide results that are not always comprehensive to farmers. The approach simplifies communication between researchers and farmers, as it ensures that researchers use terminologies that farmers understand to interpret results of conventional soil testing.

### 2.3 Assessing farmers' observed impacts of SWC on soil health

Farm management records provided by the local agricultural extension officer were examined to identify specific SWC practices across the study area. *Fanya juu* terracing stabilized with Guatemala grass (*Tripsacum andersonii*) strips across slopes, addition of farmyard manure and incorporation of crop residues in soil were the main SWC practices identified (Table 1). These SWC measures were either practiced separately or in combination with each other, which resulted in five different land management types (Table 1). To assess the impacts of different land management types on soil health, six farmers that practiced each of the five identified management types (Table 1) were randomly selected from the farm records of the area. A total of 30 farmers (six farmers from each land management type) were selected and interviewed, following ethics approval from the University of Leeds (Reference: AREA 18–044), and obtaining informed consent from research participants. Open‐ended questions (Appendix S1) on what indicators of healthy soils are and the observed changes (if any) in mentioned indicators due to specific land management practices were conducted with selected farmers between August and September 2019. Farmers were also asked the duration of their current land management practices. Interviews were conducted in Kiswahili on farmers' fields, where soils could be observed and described by farmers. Participant responses were recorded in notebooks and translated into English by two Tanzanian researchers. Translated responses were organized in spreadsheets, and technical analogues of farmers' descriptions of soil attributes were used for further data analysis (Eze et al., 2021). For example, soil erosion was used in place of ‘surface soil is washed’ or ‘presence of rills and gullies in farm’.

**TABLE 1:** Description of land management types in the study location and duration of practice

S/N	Land management code	Land management description	Crops grown	Duration of practice (years)
1	CTR—control or traditional practice with no SWC measures	Traditional practice of tilling the soil with hand hoes prior to planting	Maize is the main crop grown, intercropped with banana and sometimes pineapple or yam	>35
2	FYM—Addition of farmyard manure	Traditional practice of tilling the soil with hand hoes prior to planting. Farmyard manure is spread on the soil surface and incorporated into the soil during tillage.	Maize is the main crop grown, intercropped with banana and sometimes pineapple or yam.	18
3	FYM + CR—Addition of farmyard manure and incorporation of crop residues in soil	Crop residues and farmyard manure are spread on the soil surface and incorporated into the soil with hand hoe. Unlike in the conventional traditional tillage system, tillage is minimized here and carried out mainly to incorporate organic materials into the soil	Maize is the main crop grown with banana and sugarcane also grown as secondary crops	14
4	Ter + FYM—*Fanya juu* terracing stabilized with Guatemala grass (*Tripsacum andersonii*) strips across slopes under agroforestry system and addition of farmyard manure	Trenches of 60–80 cm (depth) by 60 cm (width) are dug 10–15 m apart across steep slopes with excavated soil thrown uphill to form bunds that are stabilized with Guatemala grass (*T. andersonii*) strips. Farmyard manure is spread on the surface between trenches and incorporated into the soil with hand hoe prior to planting	Clove, cardamom or cinnamon are the main crops with banana and sometimes sugarcane grown as secondary crops	2
5	Ter + FYM + CR—*fanya juu* terracing stabilized with *T. andersonii* strips across slopes under agroforestry system, addition of farmyard manure and incorporation of crop residues in soil	Trenches of 60–80 cm (depth) by 60 cm (width) are dug 10–15 m apart across steep slopes with excavated soil thrown uphill to form bunds that are stabilized with Guatemala grass (*T. andersonii*) strips. Crop residues and farmyard manure are spread on the surfaces between trenches and incorporated into the soil with a hand hoe prior to planting	Clove, cardamom, cinnamon or black pepper are the main crops grown with banana and sometimes sugarcane or sweet potatoes grown as secondary crops	2

*Note*. SWC = soil and water conservation. Number of farmers per land management type = 6.

### 2.4 Soil sampling

Soil sampling was conducted during the last week of August 2019, which corresponded with the harvest season for spices and planting of second round of seasonal crops e.g. maize and cassava. Soil samples were collected from the fields of 30 interviewed farmers. For each land management type, the fields of six interviewed farmers were sampled which served as replicates of the five land management types described in Table 1. Five soil samples were randomly collected with an Edelman auger from each farmer's field at 0–15 cm and 15–30 cm depths, and the five samples from each depth bulked into a composite sample for determination of particle size distribution, aggregate stability, pH, organic carbon (C), total nitrogen (N), available phosphorus (P), exchangeable basic cations (calcium, Ca; magnesium, Mg; potassium, K; and sodium, Na). Undisturbed soil cores (5 cm diameter × 5 cm height) were taken from each farmer's field at 5–10 cm and 20–25 cm, to represent an average for 0–15 cm and 15–30 cm soil layers, for the determination of bulk density and moisture retention characteristics. All the soil samples collected were transferred to the Central Soil Laboratory of the Tanzanian Agricultural Research Institute, Mlingano Centre for analysis.

### 2.5 Soil bulk density and pore size classification

The undisturbed soil cores were saturated and water retention was measured at 0, −2, −6, −10, −30, −80, −610 and −1500 kPa matric potentials. A 5‐bar pressure plate (1600 model; Soilmoisture Equipment) was used to subject soil cores to matric potentials between 0 and −100 kPa whereas a 15‐bar pressure plate (1500 model, Soilmoisture Equipment, USA) apparatus was used to subject soils to −610 and −1500 kPa matric potentials. At each matric potential, soil cores were allowed to drain until constant weight. At the end of the moisture extraction at −1500 kPa, cores were oven‐dried at 105°C for 24 hr and the volumetric moisture content at each matric potential calculated as mass loss on drying. Bulk density was also calculated as the ratio of the weight of the oven‐dried soil core to the volume of the core.

The approach described by Eze et al. (2020) was used for the determination of soil water retention curve and pore size classification. A statistical non‐linear regression was used to fit the parameters of the van Genuchten (1980) equation (Equation 1) to the measured volumetric water contents at each value of matric potential.

(1) $$\theta =\theta_{r}+\frac{\theta_{s}-\theta_{r}}{{\left[1+{\left|\alpha \psi \right|}^{n}\right]}^{m}}\;$$

*θ* is the volumetric water content (m^3^m^‐^^3^), *θ*_*r*_ and *θ*_*s*_ are residual and saturated soil volumetric water contents respectively (m^3^m^‐^^3^), and *ψ* is the soil water matric potential (cm). The parameter *α* is the inverse of the air entry potential (cm^−1^), and *n* and *m* are dimensionless parameters associated with pore size distribution. Parameter *m* was derived using the Mualem (1976) restriction:

(2) $$m=1-\frac{1}{n}\;$$

Soil water retention parameters were derived for each farmer's field and soil depth, and best fit parameter values for *θ*_*r*_, *α* and *n* were based on the smallest residual error between measured and calculated values for *θ*.

Total porosity was assumed to be equal to the volumetric water content of the soil at saturation, which was calculated as mass loss on drying saturated soil cores at 105°C to constant weight. The Kelvin equation (Equation 3) was used to determine effective pore sizes.

(3) $$d=\frac{4\text{γcosα}}{\mathrm{pgh}}\;$$

*Where: d* is the equivalent pore diameter (m), *h* is the matric potential (m), γ is the surface tension of water (72.75 mJ m^−2^), α is the pore‐water contact angle (taken to be zero), *p* is water density (0.998 Mg m^−3^) and *g* is the gravitational acceleration (9.8 m s^−2^). Following the method (Equation 4) used by Gregorich and Carter (2007), the diameter (*d*) of the smallest pore drained at a specific matric potential was calculated.

(4) $$d\;\left(\mathrm{\mu m}\right)=\frac{297.5}{\text{Matric potential}\;\left(\mathrm{kPa}\right)}\;$$

Based on differences in water retention and transport characteristics (Hayashi et al., 2006), the following four pore size classes were determined: transmission pores (>60 μm effective diameter) that hold water so loosely that it drains freely and unavailable to plants, coarse storage pores (10–60 μm effective diameter) that hold water strongly enough to be easily available to plants, fine storage pores (0.2–10 μm effective diameter) that hold water with greater force than coarse storage pores making it less available to plants and residual pores (<0.2 μm effective diametre) that hold water so tightly that it is unavailable to plants. The effective pore diameters used for classification correspond to the matric potential values of −5, −30 and −1500 kPa. As soil moisture retention was not measured at −5 kPa, the predicted values were used for determining the corresponding *d* values. The difference in volumetric water contents at matric potential values of −33 and −1500 was used as plant available water capacity (PAWC).

### 2.6 Determination of other soil physical and chemical properties

The bulk soil samples collected with auger were air‐dried and passed through an 8 mm sieve prior to analysis. To determine soil aggregate stability, aggregate size fractions were separated using the wet‐sieving method (Kemper & Rosenau, 1986). A proportion (50 g) of sieved soil samples was placed on top of a stack of sieves of different mesh sizes (i.e. 4.7, 2, 1, 0.5, 0.212 mm). The sieves were immersed in water and the soil sample on the top sieve (i.e. 4.7 mm sieve) allowed to slake for 5 min in a recipient bucket, before being moved up and down with a stroke length of 3 cm at the rate of 35 strokes min^−1^ for 2 min. The aggregates remaining on each sieve and the residual soil that passed through the 0.212 mm were oven‐dried at 40°C until constant weight and weighed. Mean weight diameter (MWD) was used as an index of aggregate stability and calculated by summing the proportion of each aggregate size fraction's weight multiplied by the mean diameter of that aggregate fraction.

(5) $$\text{Mean weight diameter}\;\left(\mathrm{MWD}\right)=\sum_{i=1}^{n}d_{i}w_{i}\;$$

*Where: d* = mean diameter of each aggregate size fraction (mm), *w* = proportion of the total sample weight, *n* = number of size fractions.

The remaining air‐dried soil samples were crushed, passed through a 2 mm sieve after visible roots and other plant materials were removed, and used for the determination of all other soil properties. The Bouyoucos hydrometer method (Gee & Bauder, 1986) was used to determine the particle size distribution of soils. Soil pH was measured in a soil suspension of 1:2.5 soil:water ratio (Robertson et al., 1999). Available phosphorus was determined using the Bray I extraction procedure (Schmidt et al., 2004) followed by spectrophotometry measurements. The Kjeldahl procedure (Nelson & Sommers, 1980) was used to determine total nitrogen. Exchangeable basic cations (calcium, Ca; magnesium, Mg; potassium, K; and sodium, Na) were extracted with 1 M ammonium acetate (Thomas, 1983) followed by flame photometry (Na and K) and ethylenediaaminetetraacetic acid‐EDTA titration (Ca and Mg). Soil organic carbon concentration was determined following the Walkley and Black method (Gillman et al., 1986), and the C stocks (Mg ha^−1^) were calculated as follows:

(6) $$\text{Soil organic}\;C\;\text{stock}\;\left(\mathrm{Mg}\;{\mathrm{ha}}^{-1}\right)=C\times \mathrm{BD}\times D\;$$

*Where*: C = Soil organic C concentration (%), BD = bulk density (g cm^−3^), D = soil depth (cm).

### 2.7 Statistical analysis

Farmers' reported impacts of the different land management types on soil health indicators were compared using Kruskal–Wallis H test with Bonferroni correction applied to pairwise comparisons. Prior to the comparison test, all the reported impacts of the five groups of land management were listed and coded (Table 2). For each type of land management, the frequency of each reported change/no change in soil health indicator was expressed as a percentage of the six farmers interviewed. Normally distributed soil parameters determined in the laboratory were subjected to a repeated measures analysis of variance (ANOVA) with depth as a within‐subject factor. The ANOVA test was used to compare the effects the different land management types and soil depth on soil organic carbon stock, aggregate stability, pore size distribution and nutrient contents. Tukey HSD post hoc test was used for mean separation.

**TABLE 2:** Kruskal–Wallis test summary with codes used for land management impacts

Soil health indicator	Coding of land management impacts	Kruskal–Wallis test statistic	Significance value
Soil colour	1 = Red to black	12.18	0.016
	2 = No change in colour		
	3 = Black to red		
Crop yield	1 = Increase	17.18	0.002
	2 = No change		
	3 = Decrease		
Soil erosion	1 = Increase	4.54	0.338
	2 = No change		
	3 = Decrease		
Soil moisture retention	1 = Increase	4.00	0.406
	2 = No change		
	3 = Decrease		

## 3 RESULTS

### 3.1 Farmers' observed impacts of climate‐smart agriculture on soil health indicators

**FIGURE 1:**
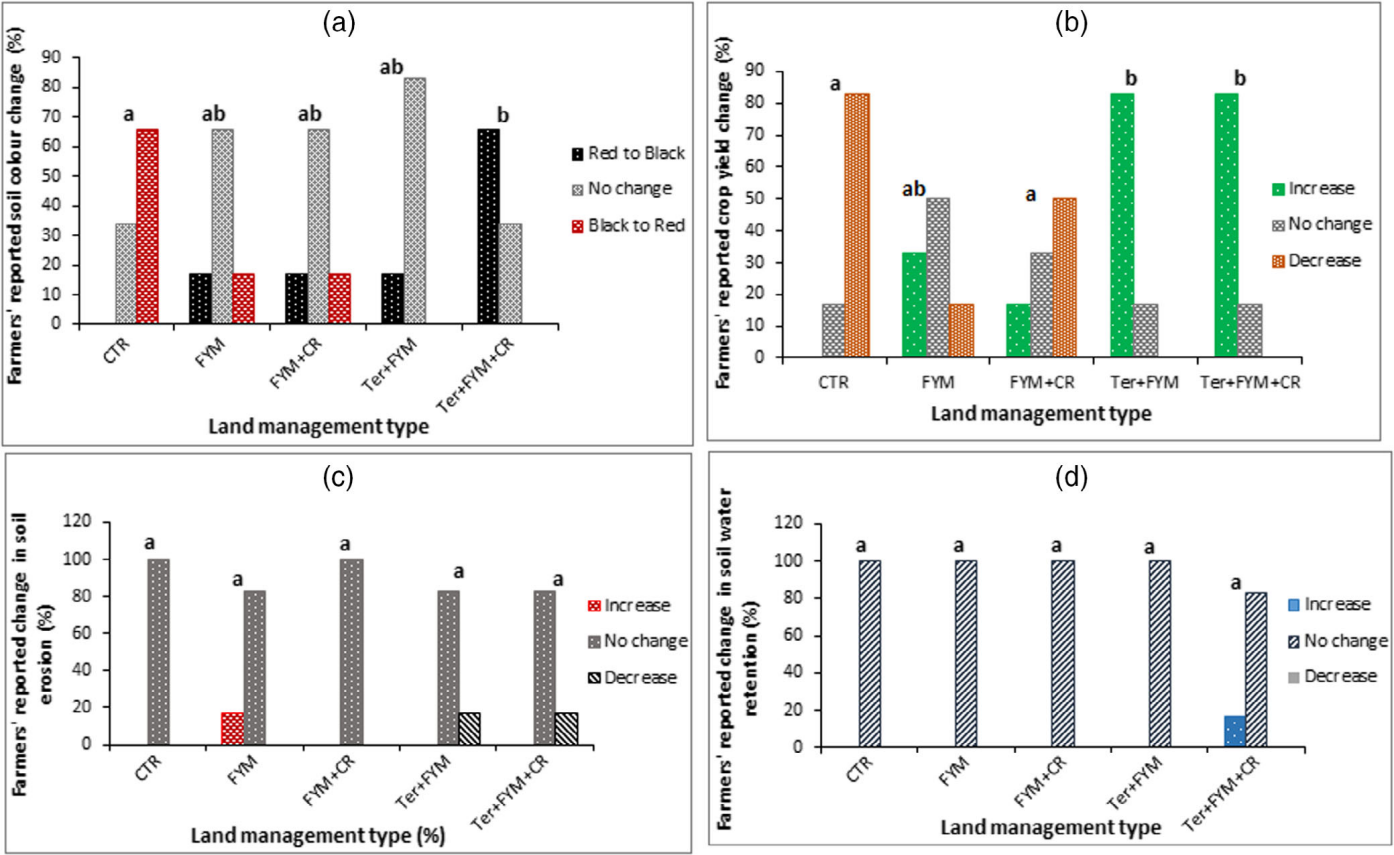
A comparison of farmers' reported impacts of different land management types on soil colour (a), crop yield (b), soil erosion (c) and soil water retention (d). Number of farmers per land management type = 6. *CTR* = control or tradition practice without soil and water conservation measures, *FYM* = addition of farmyard manure, *CR* = incorporation of crop residues in soil, *Ter* = *fanya juu* terracing stabilized with Guatemala grass (*Tripsacum andersonii*) strips across slopes under agroforestry system. Group of bars with different letters within each chart represent a statistically significant difference in the impacts of land management types on the soil health indicator presented in the chart [Colour figure can be viewed at wileyonlinelibrary.com]

Farmers in the Tanzanian EUM study villages reported changes in four soil health indicators – soil colour, crop yield, soil erosion and moisture retention due to implemented CSA practices (Table 2). However, statistically significant (*p* < 0.05) changes were reported for only soil colour and crop yield (Table 2). Out of the five land management types investigated, only the *fanya juu* terracing combined with both farmyard manure addition and incorporation of crop residues into the soil (*Ter + FYM + CR*) differed significantly from the traditional practice without any of the CSA practices (*CTR*) (Figure 1a). Under the *Ter + FYM + CR* practice, 66% of farmers reported soil colour change from red to black with the remaining 34% reporting no observed change in soil colour.

Compared with the *CTR* practice, only the *Fanya juu* terracing plus farmyard manure addition (*Ter + FYM*) and the *Ter + FYM + CR* practice had significantly different effects on crop yield (Figure 1b). For each of the two land management types with *fanya juu* terracing (*Ter + FYM* and *Ter + FYM + CR*), 83% of farmers reported an increase in crop yield with only 17% of farmers reporting lack of observed land management‐induced change in crop yield. Farmers' reports on yields were qualitative descriptions as the main crops in some of the farms have changed since the introduction of CSA practices (Table 1).

The majority of farmers (83%–100%) reported no observed change in soil erosion and soil moisture retention between all the five land management types considered (Figure 1c,d). A small proportion of farmers (17%) reported a reduction in soil erosion and an increase in soil water retention where *fanya juu* terracing was combined with farmyard manure and crop residue treatments, but these observed changes were not statistically significant (*p* < 0.05).

### 3.2 Impacts of climate‐smart agriculture on soil health indicators based on conventional soil testing

**FIGURE 2:**
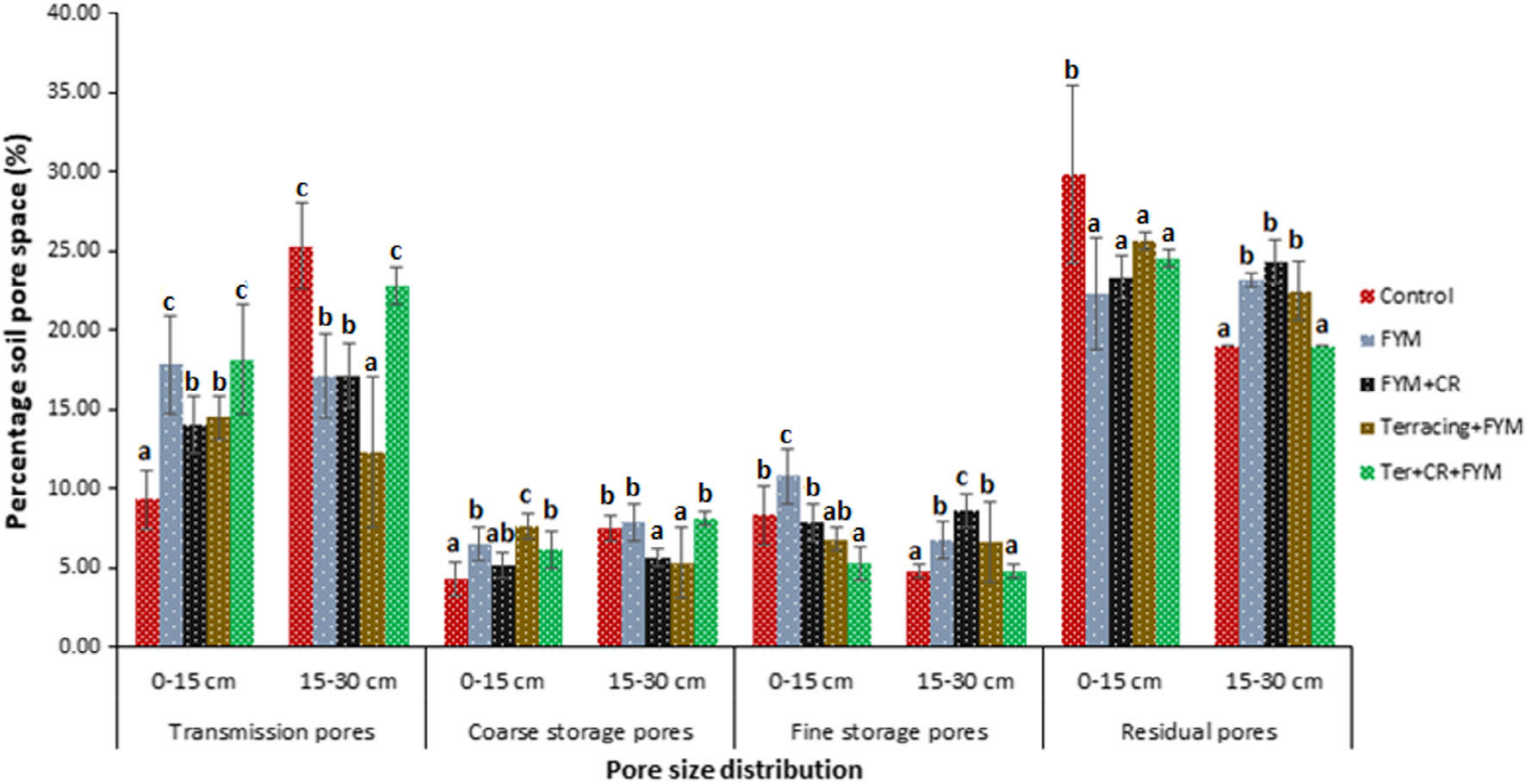
Effects of different land management types on soil pore size distribution. Control = tradition practice without soil and water conservation measures, FYM = addition of farmyard manure, CR = incorporation of crop residues in soil, Ter = *fanya juu* terracing stabilized with Guatemala grass (*Tripsacum andersonii*) strips across slopes under agroforestry system. Coloured and patterned bars represent mean percentage pore space (*n* = 6). Error bars represent ± standard deviation. Bars with different letters within the same depth and pore size differ significantly at 5% probability level [Colour figure can be viewed at wileyonlinelibrary.com]

Land management practices in the EUM study locations had statistically significant impacts on soil pore size distribution and PAWC, with significant differences in impacts between the surface (0–15 cm) and sub‐surface (15–30 cm) soil layers (Figure 2; Table 3). Out of the four SWC practices investigated, only *FYM* treatment had significantly greater PAWC (11%) than the *CTR* (PAWC = 8%) at 0–15 cm soil layer. Relative to *CTR* with PAWC of 5% at 15–30 cm soil depth, *FYM, FYM + CR* and *Ter + FYM* had significantly greater PAWC (7–9%) (Table 4). The *Ter + FYM* and *Ter + FYM + CR* systems had higher MWD (aggregate stability) than the *CTR*, but this was not statistically significant.

**TABLE 3:** Analysis of variance summary for soil physical and chemical parameters

Chemical soil parameters	Statistical test summary			Physical soil parameters	Statistical test summary		
	Factors	*F*‐value	Significance value		Factors	*F*‐value	Significance value
Soil organic carbon (SOC) concentration (%)	Mgt	1.38	0.27	Bulk density (Mg m^−3^)	Mgt	2.20	0.10
	Depth	31.07	**<0.01**		Depth	1.91	0.18
	Mgt × Depth	0.89	0.48		Mgt × Depth	0.92	0.47
SOC stock (Mg ha^−1^)	Mgt	1.97	0.13	MWD (mm)	Mgt	2.25	0.92
	Depth	29.99	**<0.01**		Depth	0.02	0.89
	Mgt × Depth	0.96	0.45		Mgt × Depth	0.92	0.89
pH	Mgt	1.21	0.33	Total porosity (%)	Mgt	0.97	0.44
	Depth	2.73	0.11		Depth	<0.01	1.00
	Mgt × Depth	0.60	0.67		Mgt × Depth	2.16	0.10
Total nitrogen (%)	Mgt	1.24	0.31	Transmission pores (%)	Mgt	10.29	**<0.01**
	Depth	5.12	**0.03**		Depth	40.29	**<0.01**
	Mgt × Depth	1.04	0.41		Mgt × Depth	23.39	**<0.01**
Available phosphorus (mg kg^−1^)	Mgt	1.81	0.16	Coarse storage pores (%)	Mgt	5.38	**<0.01**
	Depth	0.50	0.48		Depth	11.46	**<0.01**
	Mgt × Depth	1.14	0.36		Mgt × Depth	11.28	**<0.01**
Exchangeable potassium (cmol kg^−1^)	Mgt	1.87	0.15	Fine storage pores (%)	Mgt	13.18	**<0.01**
	Depth	20.84	**<0.01**		Depth	19.06	**<0.01**
	Mgt × Depth	0.26	0.90		Mgt × Depth	8.18	**<0.01**
Exchangeable calcium (cmol kg^−1^)	Mgt	1.38	0.27	Residual pores (%)	Mgt	2.76	0.05
	Depth	15.73	**<0.01**		Depth	36.29	**<0.01**
	Mgt × Depth	0.30	0.87		Mgt × Depth	14.09	**<0.01**
Exchangeable magnesium (cmol kg^−1^)	Mgt	1.67	0.19	PAWC (%)	Mgt	16.37	**<0.01**
	Depth	10.45	**<0.01**		Depth	23.97	**<0.01**
	Mgt × Depth	0.24	0.91		Mgt × Depth	10.79	**<0.01**

*Note*: Values in bold indicate factors that had statistically significant effects. Abbreviations: Mgt, land management; MWD, mean weight diameter.

**TABLE 4:** Mean ± standard deviation of soil organic carbon stock (*n* = 12), mean weight diameter (*n* = 12) and plant available water capacity (*n* = 6) under different land management practices

Soil property	Land management	Soil depth		
		0–15 cm	15–30 cm	0–30 cm
SOC stock (Mg ha^−1^)	Control			37.92 ± 13.41a
	FYM			44.88 ± 14.01a
	FYM + CR			47.55 ± 9.53a
	Ter + FYM			56.32 ± 19.53a
	Ter + CR + FYM			39.40 ± 15.93a
Mean weight diameter (mm)	Control			1.44 ± 0.29a
	FYM			1.41 ± 0.28a
	FYM + CR			1.34 ± 0.33a
	Ter + FYM			1.71 ± 0.23a
	Ter + CR + FYM			1.51 ± 0.36a
Plant available water capacity (%)	Control	8.0 ± 1.4c	4.8 ± 0.4a	
	FYM	10.7 ± 1.6d	6.7 ± 1.2b	
	FYM + CR	7.7 ± 1.0bc	8.5 ± 1.0c	
	Ter + FYM	6.3 ± 0.5ab	6.5 ± 2.3b	
	Ter + CR + FYM	5.0 ± 1.1a	4.2 ± 0.4a	

*Note*. Control = tradition practice without soil and water conservation measures, FYM = addition of farmyard manure, CR = incorporation of crop residues in soil, Ter = *fanya juu* terracing stabilized with Guatemala grass (*Tripsacum andersonii*) strips across slopes under agroforestry system. Columns with different letters within the same depth and soil property differ significantly at 5% probability level.

Across the two soil layers (0–15 and 15–30 cm), the distribution of soil pore sizes was in the order of residual pores (<0.2 μm effective diameter) > transmission pores (>60 μm effective diameter) > storage pores (0.2–60 μm effective diameter) (Figure 2). All the SWC practices investigated (i.e. *FYM*, *FYM + CR*, *Ter + FYM*, and *Ter + FYM + CR*) had significantly lower proportion of residual pores and higher transmission pores than the traditional land management system (*CTR*) at the surface 0–15 cm soil depth. All the SWC practices except *Ter + FYM + CR* had higher proportion of residual pores and lower proportion of transmission pores than the *CTR* at the 15–30 cm soil depth. The farms under farmyard manure (*FYM*) treatment had significantly higher proportions of both coarse (10–60 μm effective diameter) and fine storage soil pores (0.2–10 μm effective diameter) at the surface 0–15 cm soil depth than *CTR* systems. At the sub‐surface soil layer (15–30 cm depth), *FYM + CR* and *Ter* + *FYM* had lower proportions of coarse storage pores and higher proportions of fine storage pores than the *CTR* systems.

All the SWC practices (except *Ter + FYM + CR* at 0–15 cm soil depth) had higher soil organic C stock than the traditional *CTR* system, but this was not statistically significant (Table 4). There were also no significantly detectable differences between the different SWC practices and *CTR* in soil pH, N, P and K contents (Appendix S2). These soil chemical properties were significantly affected only by soil depth (Table 3), with greater values in the surface soil layer (0–15 cm) than in the sub‐surface layer (15–30 cm) (Table 4; Appendix S2).

## 4 DISCUSSION

### 4.1 Changes in soil properties and functions due to implemented soil and water conservation practices

Findings from this study show that the SWC practices in the Tanzania EUM study area (Table 1) led to changes in soil properties and functions (Table 3) that are indicative of soil health improvement and that farmers in this area have observed some land management impacts (Table 2). This corroborates reports from other regions of the African highlands such as the central Gojjam Highlands in Ethiopia (Adgo & Akalu, 2010) where SWC practices improved soil health leading to significant increase in crop productivity.

The main changes that the farmers attributed to SWC practices were soil colour change from red to black and an increase in crop yield (Figure 1). Soil colour is a proxy for soil organic matter and nutrient contents and in the EUM where soils are generally red due to heavy leaching and the predominance of low activity clay minerals with low nutrient retention capacity, dark/black soil colour indicates higher organic matter and fertility (Eze et al., 2021). However, the results of conventional soil testing showed no significant differences in soil C stock and nutrient content between the plots under SWC practices and those without SWC (Table 4). Soil pore size distribution and water retention were the only soil properties significantly affected by the implemented SWC practices in the EUM study area (Figure 2), which suggests that the increase in crop yield reported by farmers could be linked to improvements in soil structural properties rather than C stock or nutrient contents. With improvement in soil structure, associated benefits such as an increase in water and nutrient retention and transport, aeration, C sequestration, microbial activities and root growth which makes the agricultural system resilient to environmental stress are expected (Banwart et al., 2019). Our findings show that there was greater volume of transmission pores in the surface soil layer (0–15 cm) of SWC plots (Figure 2) which is important for reducing surface runoff and increasing the infiltration of water into the soil. The plots under SWC practices also had greater volume of coarse storage pores in the surface layer as well as greater volume of fine storage pores and residual pores in the sub‐surface layer (15–30 cm), suggesting the tendency for water that infiltrates the soil to get retained within the root zone. Enhancing infiltration and water availability within the root zone helps to mitigate the impacts of dry spells on crop productivity and ensures that crops thrive even through dry spells (Makurira et al., 2009). However, as yet only small benefits in terms of water retention have so far been realized in the EUM study area because the values of PAWC were still below 15% which is not optimum for maximum root growth (Cockroft & Olsson, 1997).

This relatively small increase in water retention matches the observation by farmers that SWC practices have not yet led to a significant increase in soil water retention due to the short duration of *fanya juu* terracing that has been implemented in the EUM study area for only 2 years. The lack of significant SWC‐induced increase in soil organic C stock and nutrient contents may also be linked to the short duration of *fanya juu* terracing. Although significant benefits of integrating multiple SWC practices including the construction of terraces, grass strips, cereal‐legumes rotation, minimum tillage and FYM application in croplands in terms of soil C sequestration can be achieved within 10 years (Namirembe et al., 2020), this may take up to 6 years (Ambaw et al., 2020).

Despite the short duration of *fanya juu* terracing, improvements in soil aggregate stability have already been recorded, with greater MWD in SWC practices with terracing than in other SWC with no terracing. Also, the farmers in the EUM observed that organic amendments such as FYM addition and incorporation of crop residues darkened the soil and increased crop yield, but greater impacts were observed when organic amendments were combined with *fanya juu* terracing. This underscores the need for combining slope barriers with other SWC and fertility management practices such as organic amendments to achieve maximum soil health benefits. Terracing has been shown to reduce surface runoff and soil loss by up to 70% (Wolka et al., 2018), highlighting its importance in the highlands with high risks of erosion (Chapman et al., 2021). With the risks of soil erosion and associated crop yield decline expected to increase significantly with climate change across SSA and especially in the highland regions (Chapman et al., 2021; Stuch et al., 2021), mainstreaming erosion‐control measures such as terracing in agricultural development strategies in the highlands are needed urgently.

A majority of farmers (83%) in the study area have observed an increase in crop yield where *fanya juu* terracing was combined with organic amendments despite the short duration of practice (Figure 1). This will provide motivation for farmers to invest efforts in maintaining such practices as they tend to favour SWC practices that deliver benefits in the short term (Meliyo et al., 2007), being unable to afford the risks and uncertainties associated with practices that deliver benefits in the long term. *Fanya juu* terracing has high initial cost of establishment with a negative net return to investment in the first 2 years after establishment (Tenge & Hella, 2005), and if not correctly and properly maintained, can lead to greater soil erosion than experienced before their construction (Ellis‐Jones & Tengberg, 2000). This high investment outlay may be a hindrance to farmers' continued use of *fanya juu* terracing despite observed benefits (Bizoza & De Graaff, 2012). This has been demonstrated in Arumeru (Kajembe et al., 2005) and West Usambara Mountains (Tenge & Hella, 2005) areas of Tanzania where farmers were more likely to adopt SWC practices with minimal labour and capital requirements. Hence, there is a need for increased government support to farmers to implement SWC that have high initial installation and maintenance costs such as *fanya juu* terracing. This will enhance farmers' adoption of sustainable land management practices, which could ensure not only increased and sustainable agricultural productivity but reduce the degradation of forest reserves in the region, currently considered a biodiversity hotspot (Bullock et al., 2014).

Considering the relatively small sample size used in this study, future studies should target a larger population of farmers in the highland region who are implementing *fanya juu* terracing and organic amendments, to strengthen the evidence base for observed benefits of such practices. Having a strong evidence base will help farmers' case for support in implementing SWC practices as well as guide regional upscaling of sustainable land management practices.

### 4.2 Benefits of combining conventional soil testing with farmers observations for sustainable land management

Farmers in the Tanzanian EUM study area observed that combining slope barriers such as *fanya juu* terracing with other SWC practices was more effective in improving soil health than organic amendments alone based on changes in soil colour and crop yield (Figure 1). This suggests that farmers are able to observe some land management impacts on soils but also require information from conventional soil testing for a comprehensive understanding of impacts that are not easily observed. As noted by Malley et al. (2009), it will be highly beneficial for land managers to integrate scientific knowledge with their local knowledge of the agro‐ecosystem. This can be achieved by improving farmers' access to relevant information about correct SWC practices and their non‐visible impacts via strengthening of the agricultural extension services and establishing strong links between farming communities and research institutions where soils can be tested and advice provided (Nyanga et al., 2016).

Hybrid approaches to soil health assessments such as the method used in this study, which combine farmers' observations with conventional soil testing, provide rich information on soil health status and give insights to the basis for land management decisions (Hermans et al., 2021). Mainstreaming such integrated approaches in local and regional soil assessment guidelines will ensure that they are used more widely. This will encourage active collaboration between farmer communities and research institutions and inform a more targeted soil assessment and the choice of appropriate land management practices.

Findings based on conventional soil testing revealed the need for enhanced soil fertility management in the EUM highlands as existing SWC practices have not increased soil nutrient contents beyond the levels in plots under conventional land management. Even in the West Usambara Mountains, soil fertility decline has been identified as a challenge to agricultural productivity (Meliyo et al., 2016; Wickama et al., 2014). Although *fanya juu* terracing, especially when stabilized with grass strips, has the potential to significantly increase soil nutrient retention, this happens in the long term of at least more than 5 years as shown by studies in the Ethiopian highlands (e.g. Adgo & Akalu, 2010). Hence, immediate measures to boost soil fertility are needed in the short term while maintaining the SWC practices with longer term benefits. Increasing the quantity of FYM or compost or combining these organic amendments with inorganic fertilizers may offer immediate soil fertility benefits, but this may have negative implications for water quality as the highlands are a major source of water to the people (Iddi, 1998). Landscape‐scale soil fertility studies to establish the right amounts of fertilizers needed to increase crop productivity without water pollution are needed in the region.

Efforts to achieve sustainable and resilient agricultural development in the Usambara Mountains have been ongoing for a long time, starting with the Usambara Mountains Development Scheme (1946–1958), the Soil Erosion Control and Agroforestry Programme in the 1960s and the Agricultural Development and Environmental Conservation Project later in the 1980s (Kikula, 1999), but there are still growing concerns for soil degradation and low agricultural productivity (Bullock et al., 2014). Hence, a farmer‐centred approach where there is an easy access to reliable soil and land management information (Kassie et al., 2015), training on correct implementation of SWC practices (Lasway et al., 2020) and reduction in the cost of SWC implementation (Nyanga et al., 2016), could be a game‐changer in the ongoing efforts to address food insecurity without degrading the environment.

## 5 CONCLUSIONS

Soil and water conservation practices in African highlands are a vital means of reversing soil degradation, increasing agricultural productivity and enhancing resilience of the agro‐ecosystem to climate shocks. Within the Tanzanian East Usambara Mountains, a combination of organic soil amendments including farmyard manure addition and incorporation of crop residues and *fanya juu* terracing led to changes in soil pore size distribution and aggregate stability that are indicative of improved soil structure. Farmers in the EUM also reported darkening of soil, a proxy for an increase in organic matter and nutrients, and an increase in crop yield due to the construction of *fanya juu* terracing combined with organic amendments. However, the results of conventional soil testing showed no significant SWC‐induced increase in soil organic carbon stock and nutrient contents, suggesting that farmers' observed increase in crop yield may be linked to changes in soil structural properties.

Findings from this study reveal two key target areas for sustainable development of the Tanzanian EUM agro‐ecosystem, which are relevant to other African highland regions. The first area is to promote a regional scale implementation of active erosion‐control measures such as *fanya juu* terracing and soil fertility management strategies with both short‐ and long‐term impacts on soil health. The second target area is to promote a farmer‐focused approach to agricultural transformation that focuses on the following priorities: (1) an integrative approach to soil assessments that combines farmers' observations with conventional soil testing for a comprehensive understanding of land management impacts; (2) strengthening of two‐way communication between farmers and research institutions for ease of access to reliable soil and SWC information; (3) strengthening of agricultural extension services and training; and (4) support to farmers to reduce the cost of SWC implementation.

## Supporting information


**Appendix S1‐S2.** Xxx

## Data Availability

The data that support the findings of this study are available from the AFRICAP Project. Data are available [from the authors with the permission of AFRICAP [https://africap.info/].
