# Supplementary material for: Assessing soil system changes under climate‐smart agriculture via farmers' observations and conventional soil testing
Source: Land Degrad Dev. 2022 May 31;33(14):2635–46. doi: 10.1002/ldr.4339 (PMC9545738; doi:10.1002/ldr.4339)
Supplement: Supplementary file 1 — Appendix S1‐S2. Xxx [file LDR-33-2635-s001.docx]

**Appendices.**

Appendix 1: Key questions that guided farmer interviews (number of farmers = 30).

| S/N | Question |
| --- | --- |
| 1 | What crops do you grow (main crop and other crops)? |
| 2 | How do you manage your field (e.g., adding manure, use of improved varieties of seeds, etc)? |
| 3 | How long is the current and previous management regimes? |
| 4 | What do you consider a good (healthy) soil? |
| 5 | How do you recognize a good (healthy) soil? |
| 6 | Have the management practices in your field caused any changes to the soil? |
| 7 | What changes in the soil have the management practices in your field caused? |
| 8 | How do you get information about the soil? |
| 9 | Do you have any other comments/questions? |

Appendix 2: Mean ± standard deviation (n=6) of selected soil physical and chemical properties under different land management practices.

| Soil property | Depth (cm) | Soil management | | | | |
| --- | --- | --- | --- | --- | --- | --- |
|  |  | Control | FYM | FYM+CR | Ter+FYM | Ter+CR+FYM |
| SOC conc. (%) | 0-15 | 2.93 ± 0.62 | 3.32 ± 1.12 | 3.14 ± 0.60 | 3.63 ± 1.22 | 2.81 ± 0.62 |
|  | 15-30 | 1.93 ± 0.73 | 2.67 ± 0.80 | 2.77 ± 0.55 | 3.13 ± 1.04 | 2.21 ± 1.04 |
| pH | 0-15 | 6.0 ± 0.1 | 6.1 ± 0.3 | 5.8 ± 0.1 | 5.7 ± 0.4 | 5.6 ± 0.6 |
|  | 15-30 | 5.8 ± 0.4 | 6.0 ± 0.4 | 5.7 ± 0.4 | 5.8 ± 0.5 | 5.5 ± 0.7 |
| Total nitrogen (%) | 0-15 | 0.21 ± 0.02 | 0.21 ± 0.04 | 0.17 ± 0.07 | 0.22 ± 0.04 | 0.19 ± 0.04 |
|  | 15-30 | 0.18 ± 0.03 | 0.18 ± 0.04 | 0.17 ± 0.02 | 0.20 ± 0.02 | 0.19 ± 0.04 |
| Available phosphorus (mg kg^-1^) | 0-15 | 14.65 ± 5.48 | 18.80 ± 9.34 | 22.36 ± 8.90 | 16.39 ± 3.71 | 15.72 ± 4.80 |
|  | 15-30 | 18.52 ± 3.19 | 18.25 ± 5.74 | 18.76 ± 2.66 | 15.94 ± 3.83 | 11.79 ± 7.08 |
| Exchangeable potassium (cmol kg^-1^) | 0-15 | 0.25 ± 0.09 | 0.27 ± 0.14 | 0.16 ± 0.06 | 0.13 ± 0.06 | 0.25 ± 0.19 |
|  | 15-30 | 0.19 ± 0.11 | 0.19 ± 0.12 | 0.10 ± 0.02 | 0.09 ± 0.03 | 0.20 ± 0.16 |
| Exchangeable calcium (cmol kg^-1^) | 0-15 | 6.28 ± 1.00 | 9.42 ± 3.91 | 5.81 ± 1.55 | 6.08 ± 3.80 | 5.04 ± 4.62 |
|  | 15-30 | 4.92 ± 3.15 | 7.90 ± 3.81 | 4.41 ± 1.57 | 5.21 ± 2.93 | 4.34 ± 4.88 |
| Exchangeable magnesium (cmol kg^-1^) | 0-15 | 1.72 ± 0.69 | 2.65 ± 1.13 | 1.49 ± 0.58 | 2.03 ± 1.36 | 1.40 ± 1.05 |
|  | 15-30 | 1.34 ± 0.60 | 2.05 ± 0.86 | 1.21 ± 0.49 | 1.61 ± 0.85 | 1.13 ± 0.97 |
| Bulk density (Mg m^-3^) | 0-15 | 1.09 ± 0.14 | 1.00 ± 0.09 | 1.11 ± 0.06 | 1.10 ± 0.07 | 1.07 ± 0.15 |
|  | 15-30 | 0.99 ± 0.05 | 1.01 ± 0.06 | 1.048 ± 0.11 | 1.13 ± 0.14 | 1.00 ± 0.06 |
| Total porosity (%) | 0-15 | 52 ± 10 | 58 ± 10 | 50 ± 5 | 54 ± 3 | 54 ± 6 |
|  | 15-30 | 57 ± 4 | 55 ± 6 | 56 ± 5 | 47 ± 11 | 55 ± 2 |
| Sand (%) | 0-15 | 39 ± 5 | 38 ± 8 | 40 ± 8 | 41 ± 5 | 41 ± 7 |
|  | 15-30 | 36 ± 7 | 35 ± 5 | 32 ± 8 | 37 ± 5 | 38 ± 5 |
| Silt (%) | 0-15 | 17 ± 2 | 14 ± 2 | 19 ± 4 | 18 ± 3 | 16 ± 4 |
|  | 15-30 | 15 ± 5 | 14 ± 2 | 20 ± 3 | 18 ± 2 | 16 ± 2 |
| Clay (%) | 0-15 | 44 ± 6 | 47 ± 8 | 41 ± 11 | 42 ± 7 | 43 ± 5 |
|  | 15-30 | 49 ± 9 | 51 ± 6 | 48 ± 10 | 45 ± 5 | 46 ± 4 |

Control = tradition practice without soil and water conservation measures, FYM = addition of farmyard manure, CR = incorporation of crop residues in soil, Ter = *Fanya juu* terracing stabilized with Guatemala grass (*Tripsacum andersonii*) strips across slopes.
